# Incidence rate of symptomatic venous thromboembolic disease in patients from a medical care program in Buenos Aires, Argentina: a prospective cohort

**DOI:** 10.1186/1477-9560-11-16

**Published:** 2013-08-01

**Authors:** Fernando Javier Vázquez, María Lourdes Posadas-Martínez, Jimena Vicens, Fernán González Bernaldo de Quirós, Diego Hernán Giunta

**Affiliations:** 1 Departamento de Medicina Interna [Internal Medicine Department], 2° piso [2nd floor], Hospital Italiano de Buenos Aires [Italian Hospital of Buenos Aires], Perón 4190 Buenos Aires C1181ACH, Argentina; 2 Internal Medicine Research Unit, Internal Medicine Department, Hospital Italiano de Buenos Aires [Italian Hospital of Buenos Aires], Buenos Aires, Argentina; 3 Epidemiology Section, Internal Medicine Department, Hospital Italiano de Buenos Aires [Italian Hospital of Buenos Aires], Buenos Aires, Argentina

**Keywords:** Epidemiology, Incidence, Pulmonary embolism, Venous thromboembolism, Venous thrombosis

## Abstract

**Background:**

The incidence of venous thromboembolic disease (VTED) is estimated to be, on average, 1–2 cases per 1,000 individuals per year worldwide. There are few data concerning the incidence rate (IR) of VTED in the Argentinean population at large.

Our aim was to estimate the IR of VTED at the Italian Hospital Medical Care Program (IHMCP) in Buenos Aires, the most populous city in Argentina.

**Methods:**

This prospective cohort study evaluated all consecutive incident cases of pulmonary thromboembolism (PTE) and deep vein thrombosis (DVT) confirmed in patients over the age of 17 who were members of the IHMCP from June 2006 to May 2012. Any patient who had an initial confirmed VTED episode and was a member of the IHMCP at the time of diagnosis was considered an incident case.

**Results:**

There were 1,138 cases of VTED for 687,871 person-years of follow-up. The crude IR of VTED was 1.65 (95% CI: 1.56 to 1.75) per 1,000 person-years. The highest IR was found in subjects >80 years old (5.92 per 1,000 person years; 95% CI: 5.41 to 6.49).

The IRs adjusted to the population of the city of Buenos Aires were 0.90 (95% CI: 0.84 to 0.95) for VTED, 0.71 (95% CI: 0.66 to 0.76) for DVT, and 0.34 (95% CI: 0.31 to 0.37) for PTE.

**Conclusions:**

VTED is a common health problem with a high IR in members of the IHMCP, especially the elderly. This is the first paper to report prospectively the cumulative incidence of VTED in Latin America.

## Background

Venous thromboembolic disease (VTED) is the most common preventable cause of death among hospital patients [1]. While VTED can affect patients of any age, it is reported that its frequency increases in older individuals [2]. The most common manifestations of VTED include pulmonary thromboembolism (PTE) and deep vein thrombosis (DVT) of the lower limbs [1,3-6]. Presentation signs and symptoms of VTED are unspecific and, when untreated, may lead to fatal PTE [7], pulmonary hypertension, and postphlebitic syndrome. [1,3-6,8,9]. The most common risk factors for the development of VTED are neoplasms, recent surgery, and hospitalisation for medical reasons [10-12].

The incidence rate for the initial episode of VTED is between 0.71 and 1.17 cases per 1,000 persons of the Caucasian population [2,13-19]. The incidence variability depends on the data source (detection through surveys or via various diagnostic studies), the record type, symptomatic or asymptomatic events, the inclusion of only primary events or also recurrences, and study design (prospective or retrospective) [2]. A number of studies have demonstrated incidence diversity in relation to race, with increased incidence rates in the Caucasian and African-American populations compared to Hispanic and Asian populations in the United States [19].

However, there are few data in Argentina and Latin America about the epidemiology of VTED [20,21] and this is the first paper reporting prospectively the incidence of VTED, DVT, and PTE in a population of Argentina. It is important to know the local epidemiology of this disease, as it can greatly impact the morbidity and mortality of patients. Thus, our goal was to estimate the incidence rate (IR) of VTED within the medical care program of the Italian Hospital of Buenos Aires, the most populous city in Argentina, using prospective symptomatic VTED data.

## Methods

### Population

A prospective cohort study was designed to evaluate all adult members of the Italian Hospital Medical Care Program (IHMCP) from June 1, 2006 to May 31, 2012. The Italian Hospital of Buenos Aires (IHBA) is a general tertiary level hospital offering comprehensive medical services to a population of 145,000 beneficiaries of the IHMCP, a prepaid health maintenance organisation (HMO) of the city of Buenos Aires in Argentina.

All medical care interventions for the beneficiaries are registered centrally in a computerised data repository, with only one electronic health record (EHR) per person. Medical care of all beneficiaries is carried out exclusively in the IHBA health system, which includes two hospitals—one of them highly specialised—with a total of 679 hospital beds. Both hospitals are located in the city of Buenos Aires and receive approximately 42,300 admissions annually. Ambulatory care is provided at these hospitals as well as 19 peripheral centres, which together receive more than 2.56 million outpatient visits per year [22].

On the other hand, according to the 2010 Argentinean National Census, the autonomous city of Buenos Aires has a population of 2,890,151 residents, of which 30% are under the age of 25 and 21.7% are over 60 years of age [23]. According to the 2010 Census demographics, the population distribution of patients included in the IHMCP is similar to that of Buenos Aires in regards to age and sex (Table 1).

**Table 1:** Comparison of the demographics of the city of Buenos Aires and the IHMCP based on the 2010 Argentina census

**Age group**	**City of Buenos Aires**		**IHMCP data**	
	**n**	**%**	**n**	**%**
0 - 14 years	472,511	16.3	21,808	14.3
15 - 24 years	395,806	13.7	13,725	9
25 - 34 years	495,663	17.2	21,087	13.8
35 - 44 years	396,202	13.7	17,975	11.8
45 - 54 years	342,647	11.9	16,303	10.7
55 - 64 years	313,251	10.8	20,569	13.5
65 years and older	474,071	16.4	40,964	26.5

Abbreviations: *IHMCP* italian hospital medical care program, *N* number, % percentage.

The study was done in compliance with the Helsinki Declaration and was approved by the Ethics Committee of HIBA (protocol N° 1877). Consent: Oral informed consent was obtained from the patient for this study, as is required in Argentina for observational designs.

### Case detection

Since 2006, the IHBA has collected VTED cases in a prospective registry (the Institutional Registry of Thromboembolic Disease, IRTD [24]) that includes all consecutive patients older than 17 years with suspected PTE and/or confirmed PTE and DVT who were diagnosed in any hospital department, including outpatient clinics, inpatient general wards for all specialties (medical and surgical), and critical care areas.

Possible VTED cases are captured for the IRTD using a computerised alert that is generated whenever the physician requests the following studies for an adult patient: computed pulmonary angiography and tomography, ventilation-perfusion scintigraphy, venous Doppler ultrasound of the lower and/or upper limbs, or angiography of the lungs and/or veins of the lower and/or upper limbs. From the possible VTED cases included in the IRTD during the 2006–2012 period, a prospective review of the EHRs was performed to confirm the presence of PTE and/or DVT. The criteria for diagnosis of VTED, DVT or PTE is presented in Table 2[25-30].

**Table 2:** Criteria for diagnosis of VTED, DVT or PTE

	**Criteria for diagnosis**
**VTED**	DVT and/or PTE
**DVT**	Confirmed with a Doppler ultrasound or angiography of the veins of the lower and/or upper limbs.
**PTE**	Positive 64-slice computed tomography (CT) angiogram (with a filling defect in the main pulmonary artery, lobar, segmental, or subsegmental branches), a high- or intermediate-probability ventilation-perfusion scintigraphy (associated with high clinical probability), and/or pulmonary angiography with evidence of a thrombus in the pulmonary artery or in the lower pulmonary arterial branch.

In this study, a case was considered incident if a patient had a confirmed first episode of VTED, PTE, and/or DVT and if the same patient was enrolled in the IHMCP at the time of diagnosis. A second episode of VTED, PTE and/or DVT in a patient was not considered incident.

### Statistical analysis

The IR was calculated for patients included in the IHMCP according to the number of incident cases of an initial VTED episode per 1,000 person-years. It was assumed that the number of new cases of VTED, DVT, or PTE corresponded to the numerator of the incidence rate and that the denominator corresponded to the number of person-years at risk. The rates were adjusted by direct standardisation to the age and sex distribution of the populations of Argentina and Buenos Aires according to the 2010 Census [23], as well as in relation to the European standard population and Segi’s world standard population [31]. In addition, the specific IRs by age and gender were evaluated. The incidence rates are expressed per 1,000 person-years, with 95% confidence intervals (95% CI), for the entire period. IRs are computed from the study data, and the cumulative incidence is estimated from the following formula using cumulative incidence as a function of t (time in years) and lambda (the IR): Cumulative Incidence (t) = 1 - exp(−lambda * t) [32].

## Results

During the study period, there were 1,138 patients with an initial confirmed VTED episode, 169 were excluded for a second episode of VTED, PTE and/or DVT. In addition, during this 6-year period, the entire cohort of IHMCP beneficiaries was followed and contributed a total of 687,871.138 person-years. Of the total number of VTED cases, 896 (78.80%) had DVT, 440 (38.66%) had PTE, and 199 (17.48%) had both PTE and DVT. Females accounted for 62.56% (712) of VTED cases, 61.38% (550) of DVT cases, and 65% (286) of PTE cases. The distribution of the risk factors in the study population were: history of cancer 39% (414/1138), pregnancy status 23% (7/31 women of childbearing potential), inpatients 33% (373/1138) and surgery 27% (310/1138).

**Figure 1:**
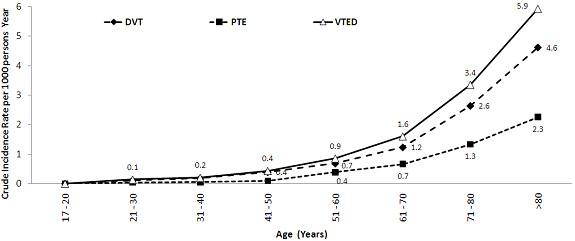
Cude IRs of VTED, DVT, and PTE by age group for the 6cyear period from 2006 to 2012.

The crude IRs per 1,000 person-years was 1.65 for VTED (95% CI: 1.56 to 1.75), 1.30 for DVT (95% CI: 1.22 to 1.39), and 0.64 for PTE (95% CI: 0.58 to 0.70) (Table 3 and Figure 1).

**Table 3:** **Crude global IRs adjusted for Argentina, Buenos Aires, Europe, and Segi’s world standard **[23,31]

	**Person-years at risk**	**Crude IR**	**Adjusted for Argentina (2010 census)**	**Adjusted for Buenos Aires (2010 census)**	**Adjusted for Europe**	**Adjusted for Segi’s world standard**
**VTED**	687,871	1.65	0.60	0.90	0.65	0.45
**(n = 1,138)**		(95% CI 1.56 – 1.75)	(95% CI 0.56 – 0.64)	(95% CI 0.84 – 0.95)	(95% CI 0.60 – 0.69)	(95% CI 0.41 – 0.48)
**DVT**	688,186	1.30	0.48	0.71	0.52	0.36
**(n = 896)**		(95% CI 1.22-1.40)	(95% CI 0.44-0.51)	(95% CI 0.66 – 0.76)	(95% CI 0.48 – 0.56)	(95% CI 0.33 – 0.39)
**PTE**	689,219	0.64	0.22	0.34	0.25	0.17
**(n = 440)**		(95% CI 0.58 – 0.70)	(95% CI 0.20 – 0.25)	(95% CI 0.31 – 0.37)	(95% CI 0.22 – 0.27)	(95% CI 0.15 – 0.19)

Incidence rates are expressed per 1,000 person-years. Abbreviations: *IR* incidence rate, *VTED* venous thromboembolic disease, *95*% *CI* confidence interval of 95%, *n* number, *PTE* pulmonary thromboembolism, *DVT* deep vein thrombosis.

The sex-specific IRs per 1,000 person-years for VTED was 1.72 (95% CI: 1.60 to 1.85) in the female group and 1.56 (95% CI: 1.42 to 1.71) in the male group; for DVT, the IRs were 1.33 (95% CI: 1.22 to 1.44) in the female group and 1.26 (95% CI: 0.13 to 1.40) in the male group; for PTE, the IRs were 0.69 (95% CI: 0.61 to 0.77) in the female group and 0.56 (95% CI: 0.48 to 0.66) in the male group. The female/male IR ratios were 1.1 for VTED (95% CI: 0.98 to 1.25), 1.05 for DVT (95% CI: 0.92 to 1.20), and 1.23 for PTE (95% CI: 1.01 to 1.50).

For cases of VTED, DVT, and PTE, stratifying the analysis by age did not identify cases among patients between 17 and 20 years of age; however, the specific rates increased with age in patients belonging to the group under study. As a result, the lowest specific IRs were found in the group of patients between 21 and 30 years of age, with values of 0.14 for VTED (95% CI: 0.08 to 0.25), 0.12 for DVT (95% CI: 0.06-0.22), and 0.03 for PTE (95% CI: 0.01-0.11). In contrast, the group of subjects over 80 years of age had the highest specific IRs, with values of 5.93 for VTED (95% CI: 5.41 to 6.50), 4.62 for DVT (95% CI: 4.16 to 5.12), and 2.25 for PTE (95% CI: 1.94 to 2.61) per 1,000 person-years (see Figure 1).

The IRs of VTED, DVT, and PTE adjusted for the population of Buenos Aires were 0.90 (95% CI: 0.84 to 0.95), 0.71 (95% CI: 0.66 to 0.76), and 0.34 (95% CI: 0.31 to 0.37) per 1,000 person-years, respectively. The IRs adjusted for the populations of Argentina, Buenos Aires, Europe, and Segi’s world standards are shown in Table 3.

The cumulative incidence rates in the follow-up period were 1.65 cases per 1,000 persons for VTED (95% CI: 1.47 to 1.85), 1.28 cases per 1,000 persons for DVT (95% CI: 1.13 to 1.46), and 0.64 cases per 1,000 persons for PTE (95% CI: 0.53 to 0.77).

## Discussion

The results of this study show that the incidence rate (IR) of symptomatic VTED in a medical care program of the Italian Hospital of Buenos Aires, remains a major and common health problem in our institution which is consistent with the findings of recent studies. There are few data in Argentina and Latin America about the epidemiology of VTED [20,21] and to our knowledge this is the first paper reporting prospectively the incidence of VTED in a population of Argentina.

Although several studies have reported the incidence of VTED in various populations [2,13,15,16], many reports included annual cumulative incidence values and not IRs. As these indicators are not equivalent, we have also reported annual cumulative incidence values to compare our findings to those of previously published studies [32].

The cumulative incidence of VTED in our study was higher than the corresponding value reported in the study of Silverstein et al. [2], as these authors detected an average age- and gender-adjusted annual incidence rate of 1.17 VTED cases per 1,000 persons in Olmsted County, Minnesota, USA. This difference could be explained by the variable clinical manifestations of the disease, the degree of certainty in the diagnosis of VTED according to the methods used to confirm the diagnosis (the 64-slice CT angiogram was not yet available between 1966 and 1990), and the study design (in contrast to the present study, the Silverstein Study was a retrospective study).

The progression of the specific IR with age was highly related to the aging of the population under study. In our study, we did not find cases of VTED in the group aged 17 to 20 years, and the study by Silverstein et al. reported only 4 cases in children younger than 15 years of age after a 25-year follow-up period. Nevertheless, both studies observed that the cumulative incidence was highest in individuals over 80 years of age.

The average annual incidence of VTED reported by Anderson et al. [13] was 1.07 per 1,000 persons in Worcester, Massachusetts, which was also lower than the corresponding value found in the present study. This difference may be due to the study design, as only inpatients were included in this study [13]. Moreover, the lack of inclusion of outpatients, who were included in the current study, may explain the differences in reported IRs because the recorded events were more frequent among outpatients than those admitted to the hospital.

In men over 50 years of age, Hansson et al. [33] reported an annual VTED IR of 3.87 cases per 1,000 person-years, which was far higher than that described in the current report. The reason for this discrepancy may be explained by the older mean age of the subjects in the Hansson study, as old age is a major risk factor for VTED. Furthermore, the inclusion of autopsies tends to overestimate the incidence due to asymptomatic PTE cases that would not have been diagnosed during patient care.

Similar to Anderson and Silverstein, we found no convincing differences between men and women with VTED [2,13] except for women younger than 55 years old, in which Silverstein et al. [2] reported a higher incidence, possibly due to hormonal influence. For PTE, we found a greater incidence among women, with a female/male IR ratio of 1.23 (95% CI: 1.01 to 1.5). In contrast, Hansson et al. [33] reported cases only in men (they excluded women from their study), and the study by White et al. [19] reported no difference between genders. In our study, the reason for our observed gender-related difference could be due to the higher proportion of women compared to men in the over-80 age group; these patients may be more vulnerable and at greater risk.

Our study shows that VTED is strongly related to age. This finding is consistent with that of White et al. [19], who demonstrated a link between the incidence of VTED and increasing age, with an exponential increase in the annual cumulative incidence from 0.05 cases per 1,000 persons in children under 15 years of age to more than 5 cases per 1,000 persons in those aged 80 years and above. Similarly, the studies by Silverstein and Anderson [2,13] reported increased VTED IRs among older patients. This observation may be related to differential exposure to clinical risk factors due to age (e.g., sedentary lifestyle, cancer, and other comorbidities) as well as a higher clinical suspicion for VTED among older patients.

The VTED “IR” adjusted for the population of Buenos Aires was 0.89 per 1,000 person-years (95% CI: 0.84-0.95), which indicates that this disease is a major health problem. The discrepancy with the crude IR of VTED in the IHMCP arises because 40% of the hospital beneficiaries in the studied population were over 55 years of age, which is higher than the proportion of the Buenos Aires city population for that age group (by approximately 12.8%). Similar findings were made for the IR of VTED in Argentina, as the value of 0.60 (95% CI 0.56 to 0.64) was lower than the corresponding aforementioned values.

Furthermore, these results are supported by data from the 2010 census, which reported that 27.2% of the population was aged 55 years or greater. This finding suggests that advanced age is strongly related to VTED. Age- and sex-adjusted IRs of DVT and PTE for the population of Buenos Aires were 0.71 and 0.34, respectively, which are also high and indicate a major health problem.

As shown in Table 3, it can be observed that all crude rates are higher than all age standardized rates to several populations, with a preserved relation between VTED, DVT and PTE rates. This is explained by the differences in age and gender distribution among the groups of people selected. Thus, IHMCP has an aged population, with higher proportion of subjects in older age strata compared to Argentina, Buenos Aires, Europe, and Segi’s world standard. Since there is no available data in Latin America on incidence of thromboembolic disease in general population, findings of this study could be cautiously generalized to other places. However, potential limitations to generalizability include aged distribution of the population of IHMCP and that it was done in only one private institution of Buenos Aires City. Therefore, future studies in other places of Latin America would increase the external validity of this study and improve the knowledge of this disease in the region.

The numbers reported here accurately depict the medical practice at the IHBA HMO. An adequate description of the situation regarding cases of VTED at the local level, both within our hospital and in the city of Buenos Aires, is essential to understand the magnitude of the problem being addressed and to identify the groups that are most at risk so that appropriate prevention measures can be implemented for the most vulnerable patients.

All detected cases corresponded to symptomatic patients who were included because of a clinically suspected initial VTED event, and these cases were recorded in the IRTD when the diagnosis of VTED was objectively demonstrated using a validated method.

A potential limitation of this study could be cases that were not considered to be included due to methodological feasibility. In this sense, we did not include autopsy findings of asymptomatic patients who died of other causes according to the attending physician and without symptoms of VTED, nor did this paper consider cases in which the physician considered the diagnosis as possible but did not confirm it due to the patient’s terminal condition as well as patients that were not studied for VTED (asymptomatic or without clinical suspicion of the attending physician). Unlike the study of Silverstein, the objective of this study was to describe only symptomatic thromboembolic events related to routine clinical practice. Other potential limitations arise from the type of study design. As a dynamic cohort study, it is possible to present a differential loss of follow up bias from subjects that leave the cohort for unknown reasons as well as an eventual variability in time exposure that cannot be addressed estimating risk by the incidence density.

## Conclusion

VTED is a major health problem, especially among the elderly. These findings have serious implications for current clinical practice because of the expected increase in the population age. To our knowledge, this is the first paper to report prospectively the cumulative incidence of VTED in Argentina and Latin America.

## Competing interests

The authors declare that they have no competing interests.

## Authors’ contributions

Conception and organization: FJV, MLPM, JV, FGBDQ, DHG, Coordination and patient data: FJV, MLPM,JV, FGBDQ, DHG, Analysis: MLPM, JV, DG, Manuscript editing: FJV, MLPM. All authors read and approved the final manuscript. All authors read and approved the final manuscript.
